# Clinical outcome in metastatic prostate cancer after primary radiotherapy

**DOI:** 10.1007/s00066-022-01993-4

**Published:** 2022-08-11

**Authors:** Matthias Moll, Harald Herrmann, Alexandru Zaharie, Gregor Goldner

**Affiliations:** Department of Radiation Oncology, Comprehensive Cancer Center, Medical University of Vienna, Währinger Gürtel 18–20, 1090 Vienna, Austria

**Keywords:** Biochemical no evidence of disease, Disease-specific survival, Overall survival, Gastrointestinal toxicity, Genitourinary toxicity

## Abstract

**Purpose:**

To describe a local radio-oncological treatment for patients with prostate cancer that metastasized to either the lymph nodes or distant regions.

**Methods and materials:**

We included 133 patients with prostate cancer that displayed either distant metastases (DM) or lymph node metastases alone (NM) and were treated between 2004 and 2019. All patients underwent computed tomography and a bone scan or 18F- or prostate-specific membrane antigen-targeted positron emission tomography. Patients received local external beam radiation therapy to the prostate to achieve local control (60–81.4 Gy to the prostate, and 45–50.4 Gy to pelvic lymph nodes), with either the 3D conformal (4-field box) or volumetric modulated arc therapy technique. A urologist prescribed additional therapy.

**Results:**

We included 51 patients with DM and 82 patients with NM. The mean follow-up was 42 months for all patients. The groups were similar in T stage, initial prostate-specific antigen, histology, androgen deprivation therapy, age, treatment techniques, and prescribed doses, but different in lymph node inclusion and follow-up times. In the NM and DM groups, the 5‑year biochemical recurrence-free rates were 52% and 24%, respectively (*p* < 0.0001); the 5‑year disease-specific survival rates were 92% and 61%, respectively (*p* = 0.001); and the 5‑year OS rates were 77% and 48%, respectively (*p* = 0.01). The groups had similar acute and late gastrointestinal and genitourinary side effects, except that late genitourinary side effects occurred significantly more frequently in the NM group (*p* = 0.01).

**Conclusions:**

DM was associated with significantly worse outcomes than NM. The long-term survival of patients with metastatic prostate cancer was low.

## Introduction

Currently, a high level of evidence supports the well-established curative therapies for primary localized prostate cancer, including watchful waiting, surgery, and radiotherapy [1–4]. However, very little evidence is available to support curative or life-prolonging treatments for patients with lymph node metastases and distant metastases. Treatment options for patients with initially positive lymph nodes include surgery, radiotherapy, and androgen deprivation therapy (ADT), or combinations [5] of these treatments. For patients with distant metastases, localized treatment options have been evaluated over the last few years. For example, McAllister et al. [6] showed that breast cancer cells could stimulate tumor growth through endocrine signaling. This mechanism was assumed to be present in a subset of renal cell carcinomas, where it was shown that a radical nephrectomy might improve survival [7]. Therefore, we reasoned that this mechanism might also apply to prostate cancer, as research showed a systemic modulation of the immune system after local radiotherapy [8].

However, the results of studies on prostate cancer have been mixed, at best. The TRoMbone trial [9], which focused on surgery for oligometastatic prostate cancer, showed acceptable quality of life after treatment, but included only 50 patients. The HORRAD trial [10] failed to prove that radiotherapy provided any benefit, and the STAMPEDE trial [11] demonstrated that radiotherapy provided survival benefits, but only to patients with a low metastatic burden, defined according to the criteria described in the CHAARTED trial [12].

The goal of the present study was to describe treatment results for patients with prostate cancer that had metastasized to the lymph node, bone, or visceral regions at the initial diagnosis, based on experiences in our department gained over the last 15 years.

## Materials and methods

This retrospective study was approved by the Ethics Review Board of our medical university, according to local laws and regulations.

Eligible patients included those treated for prostate cancer at our Department of Radiation Oncology between 2004 and 2019. We included all patients with pelvic lymph node metastases and/or distant metastases. Patients were treated locally with external beam radiation therapy. Radiotherapy was administered to achieve local control, in patients with cancer-positive pelvic lymph nodes or osseous metastases that were curatively treated, or to achieve aggressive palliation, and to prevent or to treat problems regarding urination. N and M staging were based on imaging with computed tomography (CT) and bone scintigraphy, for all patients, and when available, with 18F positron emission tomography (PET) or prostate-specific membrane antigen–PET.

The clinical target volume (CTV) was defined, based on CT and magnetic resonance imaging (MRI) scans, for planning treatments. The CTV included the prostate and the complete seminal vesicles. The total prescribed equivalent doses were delivered in 2‑Gy fractions (EQD_2Gy_) and ranged from 60 to 81.4 Gy. Pelvic lymph nodes were irradiated with a daily dose of 1.8 or 2 Gy up to 45–50.4 Gy. The dose was prescribed to cover 95% of the planned target volume, according to reports from the international commission on radiation units and measurements (reports 62 and 83) [13, 14]. Due to the long timeframe of our study, safety margins differed, depending on the radiotherapy modality. The safety margins were 5 mm, when defined with gold markers; 7–10 mm, when the volumetric modulated arc therapy (VMAT) technique was used; or 15–20 mm [15], when 3D conformal radiotherapy was used without gold markers.

Briefly, all irradiations were performed with the patient in the supine position. Patients received a rectal balloon [16] and were instructed to drink 250 ml water, 30 min prior to irradiation. Radiation was delivered with a 3D conformal 4‑field box, with the intensity-modulated radiotherapy (IMRT) technique, or with the VMAT technique.

Follow-ups were scheduled at 3 and 12 months after treatment, then annually. Biochemical failure was defined with the Phoenix criteria (a rise in prostate-specific antigen [PSA] ≥ 2 ng/ml above the nadir) [17]. During each follow-up, the physician recorded the most recent PSA value, and any late gastrointestinal (GI) and genitourinary (GU) side effects, evaluated according to Radiation Therapy Oncology Group (RTOG) grading [18]. Survival data were collected from the local death registry.

ADT and/or systemic treatment was administered by the treating urologist.

Statistical analyses were performed with GraphPad Prism 8 (GraphPad Software, San Diego, CA, USA) and SPSS (version 26, IBM, Armonk, NY, USA). A *p*-value < 0.05 was considered statistically significant. Biochemical no evidence of disease (bNED) rates were estimated with the Kaplan–Meier method. The resulting curves were compared with the log rank test. Multivariable Cox regression models were created that included the T stage (1a–c and 2a/X, 2b/c, and 3 or more), according to National Comprehensive Cancer Network (NCCN) guidelines [2], the N and M stages, age, the initial PSA value (log transformed), the Gleason score (≤ 6 = histograding 1, 7 = histograding 2, and 8–10 = histograding 3), and the applied dose, expressed in Grey units (Gy). The EQD_2Gy_ was calculated with an α/β value of 1.5 Gy. Side effects were analyzed with the Mann–Whitney U test.

## Results

Patient characteristics are provided in Table 1. The major differences between groups included the follow-up, which was shorter in the group with distant metastases, and the percentage of included lymph nodes. We treated patients with only positive metastasis to the lymph nodes for local control and patients with primary metastasis for aggressive palliation of GU symptoms. Among the patients with distant metastases, 25 (49%) also had positive lymph nodes. The median follow-ups were 16 months and 34.5 months in the distant metastases and lymph node metastasis-only groups.

**Table 1:** Patient characteristics

	NM		DM		Total	
*T stage*	82	–	51	–	133	–
T1a–2a	12	15%	5	10%	17	13%
T2b/c/X	14	17%	5	10%	19	14%
T3a/b/X	46	56%	31	61%	77	58%
T4	10	12%	10	20%	20	15%
*Gleason score*						
≤ 6 or Histograding 1	12	15%	6	12%	18	14%
7 or Histograding 2	14	17%	10	20%	24	18%
8 or Histograding 3	23	28%	16	31%	39	29%
9	24	29%	14	27%	38	29%
10	3	4%	3	6%	6	5%
Unkown	6	7%	2	4%	8	6%
*PSA*						
Min	0.6	–	2.3	–	0.6	–
Max	551.0	–	617.0	–	617.0	–
Median	28.0	–	47.3	–	31.7	–
*Age*						
Min	50	–	51	–	50	–
Max	91	–	91	–	91	–
Median	71	–	71	–	71	–
*ADT*	79	96%	51	100%	130	98%
*Technique*						
3D-conformal	54	66%	29	57%	83	62%
IMRT or VMAT	28	34%	22	43%	50	38%
Inclusion of LN	75	91%	38	75%	113	85%

*NM* lymph node metastases only, *DM* distant metastases, *PSA* prostate-specific antigen, *ADT* androgen deprivation therapy, *IMRT* intensity-modulated radiotherapy, *VMAT* volumetric modulated arc therapy, *LN* lymph nodes

Among the 133 patients included, 101 received conventionally fractionated radiotherapy to the prostate with single doses of 1.8–2 Gy and total doses of 60–81.4 Gy. The median dose was 73 Gy. Hypofractionated doses were delivered to 33 patients. Single doses ranged from 2.2 to 3 Gy. Total doses varied between 60 Gy (with 3‑Gy single doses) and 76.8 Gy (with 2.4-Gy single doses). Thus, the maximum dose (76.8 Gy/2.4 Gy) equaled an EQD_2Gy_ of 85.58 Gy, based on an α/β ratio of 1.5. Pelvic lymph nodes were irradiated with single doses of 1.8–2.5 Gy. Total doses ranged from 30 to 55 Gy. Thirteen patients received a lymph node boost, with total doses of 50–63.4 Gy. The median doses to the prostate were 74 Gy, for patients with only positive lymph nodes, and 70.6 Gy, for patients with distant metastases.

**Fig. 1:**
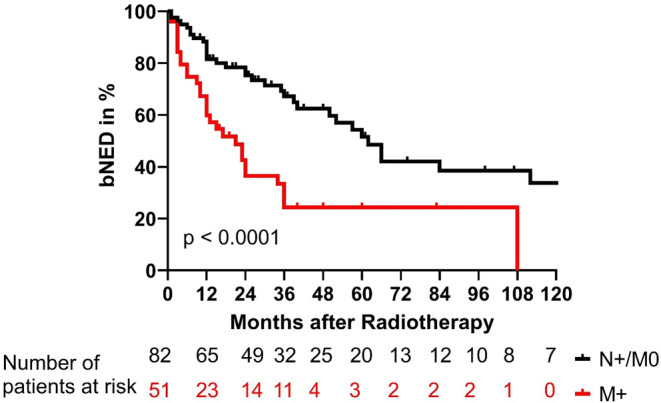
Biochemical control rates for patients with metastatic prostate cancer treated with local radiotherapy. Patients were stratified for those with only affected lymph nodes (N+/M0, *black*) and those with distant metastases (M+, *red*; *p* < 0.0001). *bNED* biochemical no evidence of disease

**Fig. 2:**
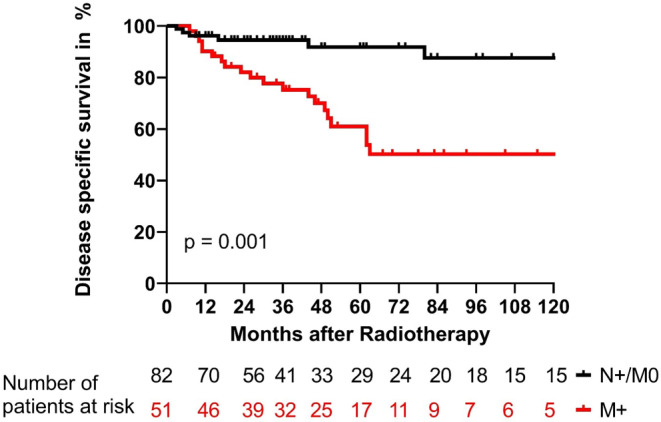
Disease-specific survival rates of patients with metastatic prostate cancer treated with local radiotherapy. Patients were stratified for those with only affected lymph nodes (N+/M0, *black*) and those with distant metastases (M+, *red*; *p* = 0.001). *bNED* biochemical no evidence of disease

**Fig. 3:**
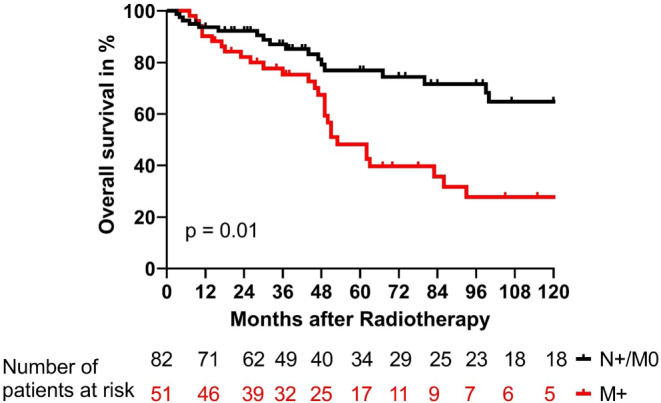
Overall survival rates of patients with metastatic prostate cancer treated with local radiotherapy. Patients were stratified for those with only affected lymph nodes (N+/M0, *black*) and patients with distant metastases (M+, *red*; *p* = 0.01). *bNED* biochemical no evidence of disease

The biochemical no evidence of disease (bNED), disease specific survival (DSS), and overall survival (OS) are shown in Figs. 1, 2, and 3. Differences in patient numbers are explained by the different sources of data acquisition: bNED rates were estimated from data gathered during the follow-up, but DSS and OS were estimated, based on all available data from all the communal hospitals in our city. After 5 years, 52% of patients without distant metastases had no biochemical failure, and only 24% of patients with distant metastases were biochemically controlled (*p* < 0.0001). The DSS rates after 5 years were 92% and 61% for patients without and with distant metastases, respectively (*p* = 0.001). OS rates at 5 years were 77% and 48% for patients without and with distant metastases, respectively (*p* = 0.01).

We also investigated the bNED, DSS, and OS, according to the dose delivered in NM patients. We compared the three dose groups (≤ 70 Gy, 70–78 Gy, and ≥ 78 Gy). No significant differences were found between groups. The *p*-values were 0.46 for bNED, 0.35 for DSS, and 0.97 for OS.

The multivariate analysis results are displayed in Table 2. We also analyzed T stage (1a–c and 2a/X, 2b/c and 3 or more) according to NCCN guidelines [2], the N and M stage, age, the initial PSA value (ln transformed), Gleason score (≤ 6 = histograding 1, 7 = histograding 2, and 8–10 = histograding 3), and the applied dose (in Gy). The EQD_2Gy_ values were calculated with an α/β ratio of 1.5 Gy. The results are displayed in Table 2.

**Table 2:** Multivariable analysis regarding bNED, DSS, and OS

	Variable	Exp(HR)	Exp(HR) (95% conf.)	*p*
*bNED*	N+	2.264	1.093–4.690	0.028
*–*	M+	4.267	2.331–7.813	< 0.001
*–*	Age	0.963	0.927–1.000	0.049
*DSS*	M+	4.342	1.632–11.555	0.003
*OS*	M+	2.375	1.267–4.453	0.007

*bNED* biochemical no evidence of disease, *DSS* disease-specific survival, *OS* overall survival, *HR* Hazard ratio, *conf.* confidence interval

Analogous to the STAMPEDE [11] and CHAARTED [12] trials, we divided our distant metastasis cohort into subgroups with low and high metastatic burdens to perform subgroup analyses. A high metastatic burden was defined as the presence of visceral metastases or ≥ 4 bone lesions, including ≥ 1 beyond the vertebral bodies and pelvis [12]. All other metastases were considered a low metastatic burden. Among the 15 patients with distant metastases, 31 (61%) had a low metastatic burden, 18 (35%) had a high burden, and 2 had an unknown burden. The vast majority of metastases was located in the bones (46 patients, 90%). In 3 cases (6%), metastases were localized in distant lymph nodes. One patient displayed peritoneal carcinosis (2%); 2 patients had liver metastases (4%). We compared bNED, DSS, and OS between the two groups and found favorable outcomes in the low metastatic burden group. These results were significant for bNED (*p* = 0.047), but not for DSS and OS (*p* = 0.08 and 0.053, respectively).

We observed no differences in acute GI or GU side effects between the groups. However, patients with distant metastases had late GU side effects significantly less frequently (*p* = 0.01), particularly grades 2 and 3, compared to patients with lymph node metastases. However, this was not the case for GI side effects. No grade 4 toxicities were observed. The complete results are displayed in Tables 3 and 4.

**Table 3:** Acute maximum gastrointestinal and genitourinary side effects

		GI			GU	
*RTOG*	NM	DM	Total	NM	DM	Total
*0*	23%	36%	28%	21%	28%	23%
*1*	44%	44%	44%	45%	46%	45%
*2*	33%	20%	28%	34%	26%	31%
*3*	0%	0%	0%	0%	0%	0%
*n*	82	50	132	82	50	132

*GI* gastrointestinal, *GU* genitourinary, *NM* lymph node metastases only, *DM* distant metastases, *RTOG* Radiation Therapy Oncology Group

**Table 4:** Late maximum gastrointestinal and genitourinary side effects

		GI			GU	
*RTOG*	NM	DM	Total	NM	DM	Total
*0*	63%	70%	66%	47%	66%	54%
*1*	20%	20%	20%	26%	24%	25%
*2*	16%	10%	14%	21%	8%	16%
*3*	1%	0%	1%	6%	2%	5%
*n*	81	50	131	81	50	131

*GI* gastrointestinal, *GU* genitourinary, *NM* lymph node metastases only, *DM* distant metastases,* RTOG* Radiation Therapy Oncology Group

Treatment intentions for patients with DM prostate cancer were curative in 18 patients (35%). In 16 cases, osseous metastases were included into the primary treatment field. In 2 cases, a singular metastasis localized on the rib was treated with a curative intention. In 33 patients (65%), treatment indication was palliative, mostly due to or to prevent problems regarding urination. Castration resistant prostate cancer at the time of radiotherapy occurred in 7 patients (14%). Details regarding ADT and further systemic treatments are listed in Table 5. It is noteworthy that some patients received multiple types of ADT and systemic treatments.

**Table 5:** ADT and systemic treatment in patients with distant metastases

ADT		Further systemic treatment	
ADT applied at any point	100%	Abiraterone acetate	22%
Goserelin	35%	Enzalutamide	16%
Leuprorelin	59%	Docetaxel	39%
Degarelix	12%	Cabazitaxel	14%
Bicalutamide	41%	Estramustine	12%
Cyproterone acetate	16%	Vinorelbine	6%
Triptorelin	6%	Radionuclide therapy	20%
Flutamide	6%	Denosumab	24%
Orchiectomy	6%	Zoledronic acid	20%
Unknown	4%	Pamidronic acid	2%

*ADT* Androgen deprivation therapy

## Discussion

Based on evidence that local cancer treatments have beneficial systemic effects [6, 7], one might assume that local treatments would also have beneficial effects in prostate cancer. However, large randomized trials either failed to show benefit, in terms of OS, like in the HORRAD trial [10], or benefits were only observed in a specific subgroup of patients with a low metastatic burden, like in the STAMPEDE trial [11]. The present study summarized our experience with local therapy for non-localized metastatic prostate cancer. Therefore, we presented data for bNED, DSS, and OS in patients with only positive nodal disease and distant metastases. We also provided data on early and late GI and GU side effects because the importance of side effects cannot be stressed enough, particularly in the palliative setting for patients with distant metastases. In addition, we surveyed the current literature on this topic (Table 6).

**Table 6:** Overview of literature for local treatment of metastatic prostate cancer

Author	Arms	Patients, *N*	Median follow-up	Primary endpoint	Result	*p*-value
Yildirim et al. [18]	RT + LK vs. NLT	44 vs. 62	14.2 months	OS	24.1 months 21.4 months	*p* = 0.08
Culp et al. [19]	7811 NLT	8185	16 months	5‑year OS & DSS	22.5% & 48.7%	*p* < 0.001
	245 RP				67.4% & 75.8%	
	129 BT				52.6% & 61.3%	
Antwi et al. [20]	7516 NLT	7858	29 months	MST	17 months	–
	222 RP				29 months	*p* < 0.0001
	120 BT				31 months	*p* < 0.0001
Parikh et al. [21]	5224 NLT	6051	22 months	5‑year OS	17.1%	–
	622 RP				–	–
	52 IMRT				45.7%	*p* < 0.01
	153 CRT				–	–
Löppenberg et al. [22]	14031 NLT	15,501	39 months	3‑year OM-free survival	50% (49–51%)	*p* < 0.001
	1470 LT				48% (47–49%)	
	77% EBRT				63% (61–66%)	
	20% RP				60% (57–63%)	
	3% BT				78% (73–83%)	
					80% (70–91%)	
Steuber et al. [23]	43 CRP	83	32.7 months	CRFS	Not specified	*p* = 0.92
	40 BST		82.2 months	OS		*p* = 0.25
Cho et al. [24]	63 NLT	140	34.0 months	3‑year OS & BCFFS	48.2% & 25%	*p* = 0.004
	77 LT				43% & 16%	(3-year OS)
	38 (27%) prostate RT				69% & 52%	*p* = 0.002
						(BCFFS)
Rusthoven et al. [25]	ADT RT + ADT	6382 5844 538	5.1 years	Median OS & 5‑year OS	55 months vs 37 months & 49% vs 33%	*p* < 0.001
HORRAD trial; Boevé et al. [9]	ADT	216	47 months	Median OS	45 months vs 43 months	*p* = 0.4
	ADT + RT	216		Median time to PSA progression	15 months vs 12 months	*p* = 0.02
STAMPEDE; Parker et al. [10]	1029 No RT	2061	37 months	Median OS	No RT: 46 months 3‑year OS 65% RT: 48 months 3‑year OS 65%	*p* = 0.266
	1032 RT			Median FFS	No RT: 13 months 3‑year FFS: 23 months RT: 17 months 3‑year FFS 32%	*p* < 0.0001
This study	RT, N+/MX	82	34.5 months	5‑year bNED	52% 19%	*p* < 0.0001
	And	–	19 months	5‑year DSS	92% 63%	*p* = 0.0005
	RT, M+	55		5‑year OS	77% 52%	*p* = 0.0037

*NLT* no local treatment, *RP* radical prostatectomy, *BT* brachytherapy, *MST* median survival time, *IMRT* intensity-modulated radiotherapy, *CRT* conventional radiotherapy, *ACM* all-cause mortality, *PCSM* prostate cancer-specific mortality, *OM* overall mortality, *CSM* cancer-specific mortality, *CRP* cytoreductive prostatectomy, *BST* best systemic therapy, *CRFS* castration resistant-free survival, *BCFFS* biochemical failure-free survival, *FFS* failure-free survival, *RT* radiotherapy, *Mx* unknown M, *M+* existent distant metastases

Some of the studies shown in Table 6 demonstrated that local therapy provided survival benefits for patients with non-localized prostate cancer. However, the two randomized trials [10, 11] showed no benefit, or benefit only in special subgroups. Therefore, local irradiation must be considered skeptically for patients with metastatic prostate cancer. However, we must also keep in mind that local irradiation or other cytoreductive therapies can have palliative effects, particularly for relieving symptoms caused by urethral stenoses.

Two major randomized controlled trials evaluated the outcome of primary radiotherapy in metastatic prostate cancer: the HORRAD trial [10] and the STAMPEDE trial [11]. The HORRAD trial included 432 patients with bone metastases who received either ADT or ADT combined with radiotherapy. Their median PSA was 142 ng/ml, and 67% of patients had more than five osseous metastases. The primary endpoint was OS, and the secondary endpoint was the time to bNED failure. No significant differences were found regarding OS, but regarding bNED, a significant difference, in favor of ADT plus radiotherapy, was observed in the 5‑year bNED. The STAMPEDE trial included 2061 men, randomized to either local radiotherapy or the standard of care. The primary endpoint was OS, and the secondary endpoints were failure-free survival, progression-free survival, metastatic progression-free survival, DSS, and symptomatic local event-free survival. They also evaluated side effects, according to RTOG criteria. In the overall cohort, OS was not significantly improved with radiotherapy (3-year OS: 65%). However, patients with a low metastatic burden benefited from local irradiation (3-year OS: 81% vs. 53%). These benefits included also significantly improved progression-free survival, metastatic progression-free survival, and prostate cancer specific survival. Taking these results into account, the STAMPEDE trial managed to highlight differences within metastatic prostate cancer. Grade 3–4 bladder and bowel acute toxicities were observed in 5% and 1% of all patients, respectively. About 2% of patients developed late grade 3 or 4 bowel or bladder toxicity. Our results were comparable to those results, with a 48% 5‑year OS in our metastatic group. However, our sample size was much smaller and included more patients with a low metastatic burden (60%) than the sample in the STAMPEDE trial (40%). However, when taking the data of the HORRAD trial [10] into account, ADT on its own leads to a median OS of 43 months, but without the side effects of radiotherapy.

When taking the large variety of distant metastatic prostate cancer into consideration, an individual treatment approach depending on the tumor load, location and comorbidities, possibly consisting of local therapy to the prostate and stereotactic body radiotherapy, as well as ADT and other systemic treatment options is not only possible, but highly recommended.

Regarding patients with clinically diagnosed lymph node metastases, the NCCN and German S3 guidelines recommend radiotherapy in combination with ADT [2, 3], or, in the case of the German guideline, also surgery [3], but evidence is sparse. Swanson et al. suggest the option of radiotherapy in this setting [5], and Horwitz et al. [19] and Bolla et al. [20] observe advantages after ADT use in patient collectives also containing some patients with clinically diagnosed node positive prostate cancer.

Among our patients with lymph node metastases, but no distant metastases, after 5 and 10 years, respectively, we observed bNED rates of 52 and 34%, DSS rates of 92 and 88%, and OS rates of 77 and 65%. Thus, our survival rates were better than those reported by Tward et al. [21], who showed 5‑ and 10-year DSS rates of 78.1 and 62.9% and OS rates of 67.8 and 44.2%, respectively. This discrepancy between studies might be explained by two main possibilities. First, the median follow-ups were quite different: 34.5 months in our study and 90 months in the Tward et al. study. This difference might account for a large part of the difference, when comparing 10-year data. Second, there may have been a difference in staging. Tward et al. enrolled patients between 1988 and 2006, and our patients were enrolled between 2004 and 2019. Therefore, better diagnostics during the later study period might have contributed to excluding some patients with primary metastases that would not have been diagnosed in the earlier study period; this difference could result in better survival rates in the later study. Especially within the first few years, good local control after lymph node metastasis-directed radiotherapy can be expected [22]. This is especially highlighted when looking at node-positive patients not receiving local therapy but only ADT, as 5‑year OS data ranges between 64 and 68% [23].

Setting aside the oncological results, it would be difficult to justify our treatment if it caused severe side effects, particularly in patients with primary metastatic prostate cancer. However, we did not observe any grade 3 or 4 GU or GI side effects, which indicated that this treatment was highly tolerable in the acute phase. Patients with positive pelvic lymph nodes showed significantly more late GU side effects. In most cases, those patients received a curative intention treatment; therefore, they underwent irradiation of the pelvic area and received higher median doses to the prostate. Around 90% of patients with distant metastases experienced late toxicities that were grade 0 or 1, at most. These results suggested that patients with an intense desire for treatment can—in most cases—be treated without major side effects, consistent with results from the STAMPEDE trial [11]. However, our short follow-up must be taken into account, when assessing the late side effects. Moreover, more than half of our patient cohort was treated with 3D-conformal radiation; thus, current treatment modalities would be expected to have fewer side effects, e.g., IMRT is known to be better tolerated than traditional treatments [24].

This study had further limitations. First, it was a retrospective study. In addition, we did not include a comparison patients group with positive lymph nodes or distant metastases that did not receive local radiotherapy. Another limitation was our short follow-up, particularly in the distant metastasis group. Moreover, the use of different irradiation techniques, doses, and safety margins could have introduced some confounding. However, dose prescriptions are also heterogenous among German-speaking centers for primary prostate cancer treatment [25]. ADT or systemic treatment was administered by the treating urologist. As patients treated in our department are referred from a large variety of hospitals, data regarding systemic therapies are largely unavailable and therefore not further mentioned in our study.

This study also had some strengths. First, it was a monocentric study; thus, there was less risk of interobserver bias, particularly regarding side effects. Second, our data reflected daily clinical practice.

## Conclusion

Patients with metastatic prostate cancer represent a population with large variations in prognosis. Nevertheless, we found that local radiation treatment was generally well tolerated. Consequently, this treatment could be considered for either a curative approach, for node-positive prostate cancer, or for palliation, without severe side effects.
